# Targeting proteostasis in multiple myeloma through inhibition of LTK

**DOI:** 10.1038/s41375-025-02682-8

**Published:** 2025-07-09

**Authors:** Thea Kristin Våtsveen, Mariaserena Giliberto, Valgerdur Bjornsdottir, Federica Centonze, Andrej Besse, Yannick Frey, Sigrid S. Skånland, Anders Tveita, Amin Alirezaylavasani, John Franklin Imbery, Kristine Misund, Veronika Reiterer, Muhammad Zahoor, Christoph Driessen, Lenka Besse, Kjetil Tasken, Fredrik H. Schjesvold, Hesso Farhan, Ludvig A. Munthe

**Affiliations:** 1 Precision immunotherapy alliance (PRIMA), Institute of Clinical Medicine, Department of Immunology, University of Oslo, Oslo, Norway; 2 KG Jebsen Centre for B cell malignancies, Institute of Clinical Medicine, University of Oslo, Oslo, Norway; 3 Department of Cancer Immunology, Institute for Cancer Research, Oslo University Hospital, Oslo, Norway; 4 Institute of Basic Medical Sciences, Department of Molecular Medicine, University of Oslo, Oslo, Norway; 5 Laboratory of Experimental Oncology, Department of Oncology and Hematology, HOCH Health Ostschweiz, Cantonal Hospital St. Gallen, St. Gallen, Switzerland; 6 Department of Biology, Faculty of Medicine, Masaryk University, Brno, Czechia; 7 Institute of Pathophysiology, Medical University of Innsbruck, Innsbruck, Austria; 8 Department of Clinical and Molecular Medicine, Norwegian University of Science and Technology, Trondheim, Norway; 9 Department of Oncology and Hematology, HOCH Health Ostschweiz, Cantonal Hospital St. Gallen, St. Gallen, Switzerland; 10 Oslo Myeloma Center, Department of Haematology, Oslo University Hospital, Oslo, Norway

**Keywords:** Myeloma, Translational research, Apoptosis

## Abstract

Multiple myeloma (MM) cells secrete high levels of immunoglobulin and are therefore addicted to mechanisms that maintain proteome homeostasis (proteostasis). While proteasome inhibitors that target the degradative aspect of proteostasis have proven effective, only limited attempts have been made to target protein secretion. Here we show that the receptor tyrosine kinase LTK is a regulatory node in the proteostasis network that responds to secretory load and helps cells maintain a high secretory output. LTK is a highly similar paralog to ALK and by repurposing existing ALK inhibitors, we demonstrate that targeting LTK causes immunoglobulin retention, ER stress and subsequent apoptosis of primary MM cells, even in patients refractory to proteasome inhibitors. Thus, LTK is a novel therapeutic target in the biosynthetic pathway of proteostasis, with significant potential for MM treatment.

## Introduction

Multiple myeloma (MM) is a plasma cell malignancy that is characterized in most cases by the excessive secretion of immunoglobulins (M-protein). Plasma cells produce thousands of immunoglobulins per second [1, 2] and the in vivo secretion rates of MM cells were estimated to be up to 85,000 molecules of IgG per cell per minute [3]. MM cells are therefore potentially addicted to mechanisms that maintain protein homeostasis (proteostasis) [4, 5]. Proteostasis involves both the synthesis and trafficking of proteins, as well as the degradation of misfolded proteins. Misfolded proteins in the endoplasmic reticulum (ER) are targeted to the proteasome to ER-associated degradation. The proteasome inhibitor (PI) bortezomib has been successfully used in myeloma treatment, leading to the subsequent approval of second generation proteasomal inhibitors, which are part of the many lines of standard of care for most myeloma patients [6, 7]. The biosynthetic and degradative parts of proteostasis are highly interconnected. However, no clinically or preclinically successful attempts have been made to target the biosynthetic part of the proteostasis network. This is partly because secretion has been considered notoriously undruggable. Identifying druggable targets within the secretion machinery holds promise not only for new therapies against MM, but also for other malignancies and conditions [8–10].

The ER is a major site for proteostasis and is equipped with various control systems that sense and respond to imbalances in the proteome, with the unfolded protein response (UPR) being the most prominent. The export of secretory proteins from the ER occurs in a COPII-dependent manner. Components of the COPII coat are not druggable, making it challenging to target ER export pharmacologically. In our previous work, we identified the receptor tyrosine kinase LTK as a regulator of COPII-dependent ER-export [11–13]. Blocking LTK function led to the inhibition of secretory protein export from the ER [14]. LTK is a paralog of anaplastic lymphoma kinase ALK [15], and is only expressed in myeloma cells following the rare event of an ALK-translocation; hence, ALK-inhibitors have not generally been used in myeloma therapies [16]. However, we previously demonstrated that ALK inhibitors blocked secretory trafficking from the ER [14]. We therefore hypothesized that targeting LTK in ALK-negative MM cells might inhibit immunoglobulin export from the ER, induce ER stress and ultimately trigger cell death. In the current study, we show that inhibition of LTK triggers cell death in a manner dependent on the secretory load of the cells. We demonstrate that LTK is expressed across different stages of MM and in various MM cell lines. Our work establishes LTK as a promising novel target for myeloma therapy.

## Materials and methods

### Patient samples and primary MM cell sample processing

MM patients were recruited from the Oslo Myeloma Center at Oslo University Hospital following signed informed consent in compliance with the Declaration of Helsinki. The study was approved by the Regional Committee for Medical and Health Research Ethics of South-East Norway (REC#2016/947 and 2012/174). Bone marrow mononuclear cells (BMMCs) were prepared from patient bone marrow aspirates using Lymphoprep^TM^ density gradient centrifugation. For MM cells, we followed a previously published prototocol [17, 18]. Briefly, CD8^+^ cytotoxic T lymphocytes were depleted by using CD8 Dynabeads (Life Technologies) and BMMCs were subsequently stimulated by expanding T helper cells in the presence of Human T-Activator CD3/CD28 Dynabeads (Life Technologies), and 100 U/ml human interleukin-2 (hIL-2, Roche, Germany). After 48 h, BMMCs were subjected to CD138^+^ enrichment to isolate MM plasma cells using MACS CD138^+^ microbeads (Miltenyi Biotec, Germany) These cells were used for drug treatment assay. For other assays CD138^+^ enrichment was performed on fresh cells that were assayed immediately.

### Drug treatment and cell viability assay

The drugs used included crizotinib, ceritinib, entrectinib, ensartinib, alectinib, brigatinib and lorlatinib (Selleck Chemicals LLC). CD138^+^ MM cells (5000–10,000 cells/well) from activation assays were tested in 384-well plates against ALK-inhibitors concentrations ranging from 0.01 to 10 µM, as previously described [18]. Drug sensitivity score (DSS) was calculated as previously described [19]. See also Supplementary Methods for further details.

### Cell lines

MM cell lines L363 and L363-BTZ were maintained in the RPMI-1640 culture medium (Sigma Aldrich, Buchs, Switzerland) supplemented with 10% heat-inactivated fetal bovine serum (FBS), 100 µg/ml streptomycin and 100U/ml penicillin/streptomycin (Sigma Aldrich, Buchs, Switzerland). The bortezomib-resistant cell lines were established and maintained from their parental cell line by continuous exposure to the drugs [20, 21]. The URVIN cell line was established from a patient by us [22], supplemented with 1 ng/ml IL-6 (R&D systems). The human myeloma cell lines (HMCLs) used for qPCR were OH-2 [23, 24], IH-1 [25], VOLIN, KJON [26], URVIN, JJN3, INA-6 [27] and U266. Cell lines has been verified using binominal fingerprint score as shown in [22], and regularly tested for mycoplasma (Lonza MycoAlert PLUS Mycoplasma Detection, BioNordika). RPMI-8226 was used for immunofluorescence. HeLa cells with inducible IgM expression has been described previously [28]. HeLa cells expressing LTK with LTK∆exon7 DNA constructs are detailed in the Supplementary Methods.

### Immunofluorescence, immunoblotting and mass spectroscopy

HeLa cells grown on glass coverslips were transfected with a construct encoding GFP-LTK∆exon7 and stained with rabbit anti-CLIMP63 (CKAP4) pAb (1:500, gift from Hans-Peter Hauri) [29]. Details for immunoblotting, immunoprecipitation and mass spectroscopy are found in the Supplementary Methods.

### Gene expression data and DNA constructs

Omics data were downloaded from the CoMMpass℠ study, IA-13 and IA-17 builds and included 767 and 921 MM samples respectively (www.themmrf.org). These data were generated as part of the Multiple Myeloma Research Foundation Personalized Medicine Initiatives (https://research.themmrf.org). RNA seq data were represented as Fragments Per Kilobase of transcript per Million mapped reads, FPKM. Data were visualized using Graphpad Prizm 10 with violin plots and FKPM < 1 was considered negative. Details on RT-PCR, site-directed PCR mutagenesis and pcDNA-LTK∆exon7 [30] are provided in the Supplementary Methods.

### ELISA

IgG positive CD138+ separated cells (1×10^6^/ml) were seeded in serum free human plasma like medium (HLPM, Thermo Fisher Scientific) with 5 µM ALK inhibitors or DMSO control for 3 h. The medium was collected, diluted two-fold, and analyzed using a ELISA total IgG kit (Invitrogen) according to the manufacturer’s instructions.

### Intracellular immunoglobulin staining with fluorescence flow cytometry

URVIN or IgG positive CD138+ separated cells (1 × 10^6^/ml) from patients were seeded in HLPM with 5 µM ALK inhibitors for 18 h (URVIN) or 3 h for primary cells. Cells were harvested, stained with Dead Cell Stain FarRed IR (Thermo Fisher Scientific), fixed with the TF fix kit (eBioscience), barcoded with Pacific Blue/Orange as previously described [31], and stained with anti-IgL PE-Cy-7 clone MHL-38 (316622, BioLegend) and/or anti IgK-AlexaFluor700 clone-G20-193 (561319, BD Pharmingen). Cells were acquired using an Attune Flow cytometer (Thermo Fischer Scientific) and data were analyzed using FlowJo 10.

## Results

### LTK is expressed in MM patients

**Fig. 1:**
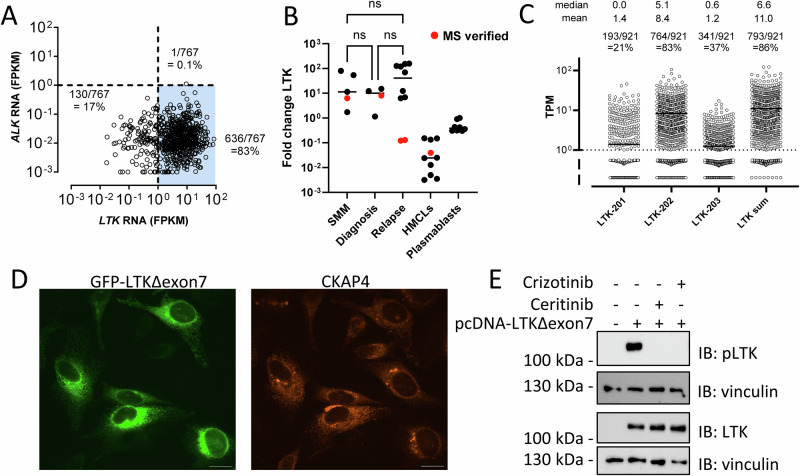
LTK, but not ALK is expressed in multiple myeloma cells. **A** *LTK* expression versus *ALK* expression from MM patients enrolled in the CoMMpass study (build IV13). FPKM: Fragments Per Kilobase Million. **B** qPCR analysis of *LTK* in smouldering multiple myeloma (SMM), newly diagnosed treatment naive MM, relapsed/refractory MM, human myeloma cell line (HMCL), and healthy donor plasmablasts. Red filled circles indicate samples tested and validated by mass spectrometry for LTK protein. One-way ANOVA, SMM vs. Diagnosis *p* = 0.8235; SMM vs. Relapse *p* = 0.4813; Diagnosis vs. Relapse *p* = 0.2111. **C** LTK transcript isoforms, LTK-201 (ENST00000263800), LTK-202 (ENST00000355166), and LTK-203 (ENST00000453182) in MM patients enrolled in the CoMMpass study (build IV17) and the sum of the three transcripts are shown. Medians, means, and fractions of transcript positive MM patients are indicated (Transcripts per million, TPM > 1). **D** Confocal micrograph of HeLa cells expressing LTK∆exon7, the dominant MM isoform of LTK (LTK-202). Left: LTK∆exon7-GFP signal (green), right: ER localization visualized through anti-Cytoskeleton-Associated Protein 4 (CKAP4) (red). Images are maximum intensity projections of several confocal sections. Scale bars: 10 μm. **E** Western blots of cell lysates from LTK∆exon7 HeLa cells or mock transfectants exposed to crizotinib or ceritinib, as indicated. Immunoblots of anti-phospho-LTK and anti-LTK with anti-vinculin as loading control are shown.

Expression of *LTK* and *ALK* was analyzed using RNA sequencing data from 767 MM patients from the CoMMpass study [32]. We found that the majority of patients (83%) had myeloma cells expressing *LTK*, but not *ALK* (0.1%) (Fig. 1A). Further analysis of LTK expression by qPCR was conducted in smouldering myeloma (SMM), newly diagnosed MM, relapsed/refractory MM, human myeloma cell lines (HMCLs), and healthy donor plasmablasts expanded from CD19^+^ peripheral blood cells. We found no significant difference in LTK expression between SMM, primary MM and relapsed MM (Fig.1B). LTK expression was also not significantly different between lines of treatment and in longitudinal samples from MM patients (Supplementary Fig. S1A, B). Moreover, MM cells showed, on average, over 10-fold higher LTK expression than healthy donor plasmablasts (Fig. 1B). Two relapsed/refractory MM patients had intermediate/low LTK levels. The HMCLs expressed low LTK, as could be expected since many HMCLs secrete relatively low levels of immunoglobulins. To confirm the expression at the protein level, cell lysates were tested for the presence of LTK by mass spectroscopy. In five out of all five samples tested, LTK peptide sequences were detected, including the MM cells from both intermediate/low LTK expressing relapsed/refractory MM patients (Fig. 1B, red symbols).

We next examined LTK expression in the CoMMpass study in more detail. The LTK gene is located on 15q15.1, but although chromosome 15 is frequently triploid in hyperdiploid myeloma patients, we did not find increased LTK expression due to chromosomal amplification (*p* = 0.4874, one-way ANOVA, Supplementary Fig. S1C), nor did we find any correlation between *LTK* mRNA levels and secreted M-protein (data not shown). However, we identified MM cells that had no or very low IgH expression (IgG1, IgG2, IgG3, IgG4, IgA1, IgA2), these had significantly less LTK expression than MM with above average IgH expression (*p* = 0.0009, Mann-Whitney, Supplementary Fig. S1D). A similar significant difference was seen in IgG1-positive MM: LTK expression in IgG1^HI^ patients with higher than average IgG1 was increased and significantly different from that found in MM with lower than average IgG1 expression (*p* = 0.027, Mann-Whitney, Supplementary Fig. S1E). A similar LTK expression was found in non-malignant bone marrow plasma cells (Supplementary Fig S1F) [33]. There were no correlations between LTK expression level and the presence of common mutations in *RAS, TP53* or *DIS3* (Supplementary Fig. S1G). Analysis of data from the CoMMpass study also provided evidence that the main isotype of LTK transcript in MM cells was the LTK-202 isoform, which lacks exon 7 (LTK-202) (Fig. 1C). To validate ER localization of this isoform, we generated GFP-tagged LTK∆exon7 transfected HeLa cells and found co-localization of the LTK∆exon7 protein with CKAP4, a known ER marker (Fig. 1D). We also confirmed LTK localization in an HMCL, RPMI-8226, and in a primary myeloma patient sample, finding it localized to intracellular ER-like membranes (Supplementary Fig. S1H, I).

As the LTK kinase domain has very high homology to ALK, we tested whether the ALK inhibitors ceritinib and crizotinib could dephosphorylate LTK∆exon7 in HeLa cells. When cells were exposed to either of these drugs, we observed a dramatic downregulation in the ratio of phosphorylated LTK (pLTK) to dephosphorylated LTK in Western blot analyses (Fig. 1E).

Taken together we confirm that myeloma cells express LTK, but not ALK, and that the main isoform of LTK in myeloma cells localizes to the ER and can be inhibited by ALK inhibitors.

### LTK plays a role for MM cells to cope with elevated secretory load

**Fig. 2:**
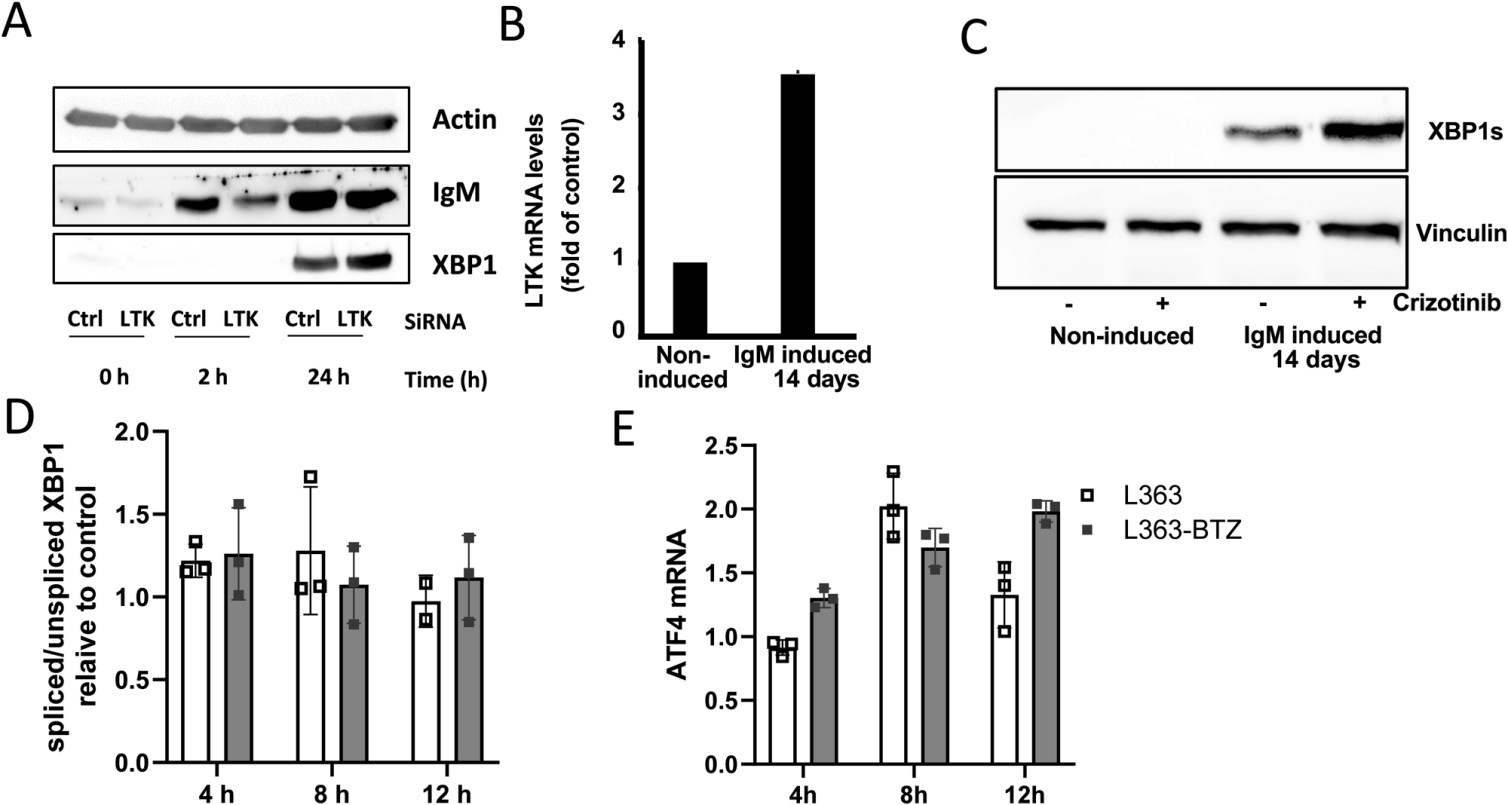
LTK allows cells to cope with high secretory load. **A** HeLa cells expressing inducible IgM (heavy and light chain) were transfected with control or LTK siRNA. After 72 h, cells were treated with mifepristone to induce IgM expression followed by lysis and immunoblotting against the indicated proteins. **B** LTK mRNA is fourfold higher in HeLa cells with 2 weeks IgM expression compared to control. **C** Immunoblot of HeLa cells with induce IgM expression over a period of 2 weeks, subsequently treated with 1 μM crizotinib for 24 h. **D** L363 and bortezomib resistant L363-BTZ cells were treated with 5 µM crizotinib and the expression of spliced vs non-spliced XBP1 was not statistically different regardless of bortezomib sensitivity (*p* =0.533) for L363 vs L363-BTZ, two-way ANOVA (see also Supplementary Fig. S2A, B for analysis of crizotinib vs no drug for each cell line). **E** ATF4 was determined 4, 8 and 12 h post-treatment. Spliced vs non-spliced XBP1 *p* = 0.533 for L363 vs L363-BTZ, as well as ATF4 *p* = 0.267 between L363 and L363-BTZ and *p* = 0.013 for ATF4 expression over time (Supplementary Fig. S2).

Since MM cells produce high amounts of immunoglobulins, we wanted to test whether LTK could mediate an adaptation to high secretory cargo load. We therefore used HeLa cells with inducible expression of secretory heavy chain of IgM [28]. Expression of the IgM heavy chain resulted in ER overload and was shown to trigger the unfolded protein response, UPR [28, 34]. Induction of IgM expression in HeLa cells for 24 h resulted in a mild activation of the UPR, as indicated by an increase in spliced X-box binding protein-1 (XBP1s) levels (Fig. 2A). When LTK expression was silenced, cells responded with higher levels of ER stress upon induction of IgM expression (Fig. 2A).

In the same model system, IgM was induced for 14 days to allow cells to mount long-term adaptive responses to this increased secretory load. In the hypersecretory cells, we found a 4-fold increase of LTK mRNA (Fig. 2B), consistent with LTK playing an important role in helping cells cope with elevated secretory load. This result aligned with the correlation between IgG1 and LTK expression in primary MM cells as described above (Supplementary Fig. S1C). The IgM-expressing cells showed only moderately increased ER stress levels (Fig. 2C). Furthermore, when LTK was inhibited by crizotinib, hypersecretory cells exhibited a 2.5-fold increase in ER stress. In comparison, the non-IgM secreting control cells showed no LTK adaptation and no ER stress regardless of crizotinib treatment (Fig. 2C).

To determine whether LTK inhibition induces ER stress in HMCL, wild-type (L363) and bortezomib-resistant (L363-BTZ) cells were treated with crizotinib. Consistent the ER stress induction shown above, crizotinib treatment induced ER stress as demonstrated by increased splicing of XBP1 and induction of ATF4 (*p* = 0.013 over time) in both wildtype and bortezomib-resistant HMCL cells (Fig. 2D, E, Supplementary Fig. S2A, B). There was no significant difference between L363 and L363-BTZ response to crizotinib (XBP1 *p* = 0.533 and ATF4 *p* = 0.267, Fig. 2D, E)). These data suggest that LTK plays an important role in helping cells cope with a high secretory load, such as immunoglobulin secretion, with a significant increase in ER stress markers upon treatment with the ALK-inhibitor crizotinib.

### Targeting LTK reduces the viability of multiple myeloma cells

**Fig. 3:**
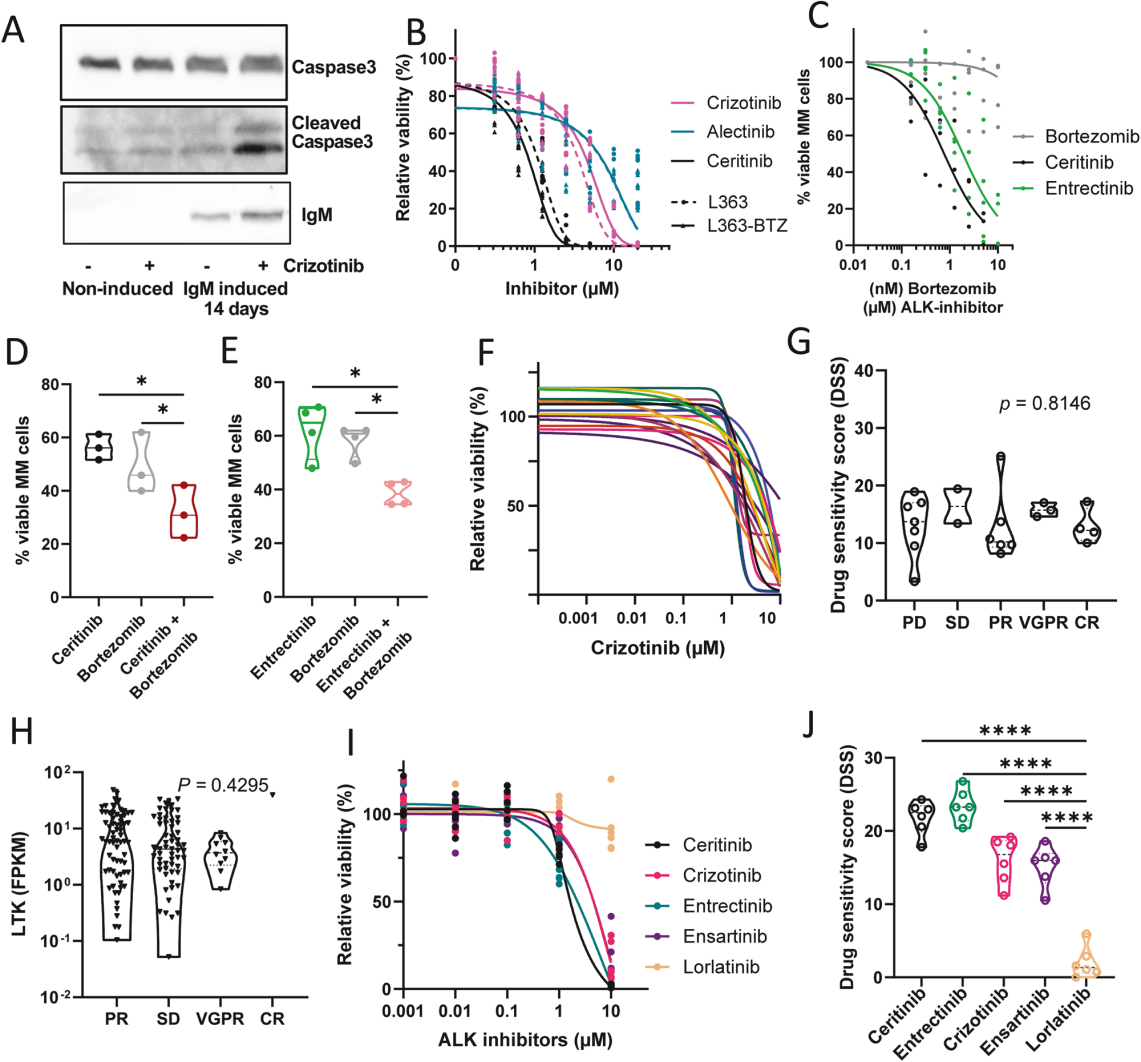
Inhibition of LTK in hypersecretory cells reduces viability. **A** Immunoblot of caspase-3 in HeLa cells with induced IgM expression over a period of 2 weeks, subsequently treated with 1 μM crizotinib for 24 h. **B** Dose-response curve for alectinib, ceritinib and crizotinib, after 24 h of treatment in L363 and L363-BTZ cells. The results represent the means and ±SD of three independent experiments using the CCK-8 assay. **C** Viability (CellTiterGlo) dose-response curve in for ceritinib, entrectinib and bortezomib after 48 h of treatment in CD138^+^ bortezomib resistant myeloma patient samples *(n* = 3). Further drug combinations are shown in Supplementary Fig. S2E. **D** Combination of ceritinib with bortezomib in CD138^+^ MM cells from bortezomib sensitive patient samples (*n* = 3). The shown data are the concentration of closest to IC50 as possible for each single drugs for each patient, and the combination of these two concentrations. A full viability (CellTiterGlo) dataset is shown in Supplementary Fig. S2F for all combinations in one representative patient. **E** Combination of entrectinib with bortezomib in CD138^+^ bortezomib sensitive patient samples (*n* = 4), as in (**D**). Patient information for (**C**–**E**) is shown in Supplementary Table S1. One-way ANOVA *p* < 0.05 (*). **F** CD138^+^ cells from 22 myeloma patients were treated with ceritinib in increasing concentrations for 72 h before viability was measured with the CellTiterGlo assay. **G** Drug sensitivity score (DSS) of crizotinb was measured in the 22 patient samples, and the samples are sorted by their response to their last treatment: progressive disease (PD), stable disease (SD), partial response (PR), very good partial response (VGPR), and complete remission (CR) (*n* = 22, 72 h *p* = 0.8146 by ANOVA summary). **H** LTK RNA expression from MM cells from the CoMMpass study (build IV17) sorted similarly as in G (*n* = 139, *P* = 0.43, ANOVA multiple comparison). **I**, **J** In the same type of drug assay as in (**F**), six additional patients were treated with ceritinib, crizotinib entrectinib, ensartinib and lorlatinib. **I** Relative viability (%) is shown as average of *n* = 6 patient samples for each drug, with three technical replicates per patient sample. **J** DSS values are shown for all six patient samples, statistical comparisons with low-activity lorlatinib are shown. One-way ANOVA *p* < 0.0001 (****). See also Supplementary Fig. S3A for individual patient drug response curves and Supplementary Tables S2, 3 for patient information related to (**F**, **G**, **I**, **J**).

Induction of ER stress in hypersecretory cells prompted us to test whether LTK inhibition would trigger cell death. In the same experiment as in Fig. 2C, we found that the ER-stressed cells with hypersecretion of IgM had cleaved caspase 3 when LTK was inhibited with crizotinib. No apoptosis was detected in non-treated cells, nor in the non-IgM secreting controls (with or without crizotinib) (Fig. 3A). Thus, the secretory load of cells determines the sensitivity to ALK inhibitors.

We next tested whether HMCL cells were sensitive to LTK inhibition. The L363 and L363-BTZ cells were treated with increasing concentrations of crizotinib, ceritinib and alectinib. We observed the same concentration-dependent cytotoxicity in both bortezomib-sensitive and bortezomib-resistant cells (Fig. 3B). This was repeated with patient samples previously treated with bortezomib, we observed a concentration-dependent cytotoxicity for ceritinib and entrectinib in all three patient samples tested (Fig. 3C). For bortezomib responsive patient samples we found a significant additive effect when comparing the closest value to IC50 in our assay for bortezomib and ceritinib (Fig. 3D) or entrectinib (Fig. 3E). Similar data from HMCLs are shown in Supplementary Fig. S2C, D.

To extend these results to primary MM cells, we tested the efficacy of crizotinib in MM cells from a cohort of patients, where 17 out of 22 patients that had become refractory to at least one proteasome inhibitor (Supplementary Table S2). In the presence of crizotinib, we found a reduced cell viability with a median IC_50_ of 1.93 µM crizotinib (Fig. 3F). Using a measure that provides a metric for the area under the curve (AUC), we calculated the drug sensitivity score (DSS) [18, 19] for the 22 MM patients, and results were sorted according to patient response to their previous treatment; progressive disease (PD), stable disease (SD), partial response (PR), very good partial response (VGPR), and complete remission (CR) derived from the International Myeloma Working group (IMWG) response criteria from 2016 [35]. There was no difference in the response to LTK inhibition between the groups (*n* = 22, *p* = 0.87) indicating that LTK inhibition had similar effect regardless of the preceding response status (Fig. 3G). In line with these results, there were no significant differences in *LTK* expression in the corresponding groups after analysis of data from the CoMMpass study (*n* = 139, *p* = 0.43), Fig. 3H.

To extend the drug effect analysis to additional ALK-inhibitors beyond crizotinib, we also tested ceritinib, entrectinib, ensartinib and lorlatinib on MM cells from six additional patients (Fig. 3I, J). With some variation between the drugs, we found that all drugs, except lorlatinib, showed a significant effect on MM cell viability (Fig. 3; see Supplementary Table 1 and 2 for patient information, and Supplementary Fig. S3A for individual response curves). Lorlatinib, which has a different chemical structure and binding site than the other ALK inhibitors and more than 38x lower cell free (IC_50_) activity for LTK than for ALK [36], resulted in only minor inhibition at 10 µM. DMSO served as negative control, and lorlatinib, as the only ALK-inhibitor with a low LTK activity, was also used for statistical comparisons. All 6 patients had received proteasome inhibitors (bortezomib or carfilzomib) in at least one line of previous treatment. ALK-inhibitors, except lorlatinib, were also effective in a range of HMCLs representing different genetic, mutational backgrounds and gene expression (Supplementary Figs. S3B, S4A, B). Additional assays with LTK inhibition of primary myeloma cells in bone marrow mononuclear cell (BMMC) cultures for 48 h are shown in Supplementary Fig. S5. In summary, we observed a decrease in viability in MM cells regardless of previous treatments when treated with the various ALK inhibitors.

### Targeting LTK reduces antibody secretion

**Fig. 4:**
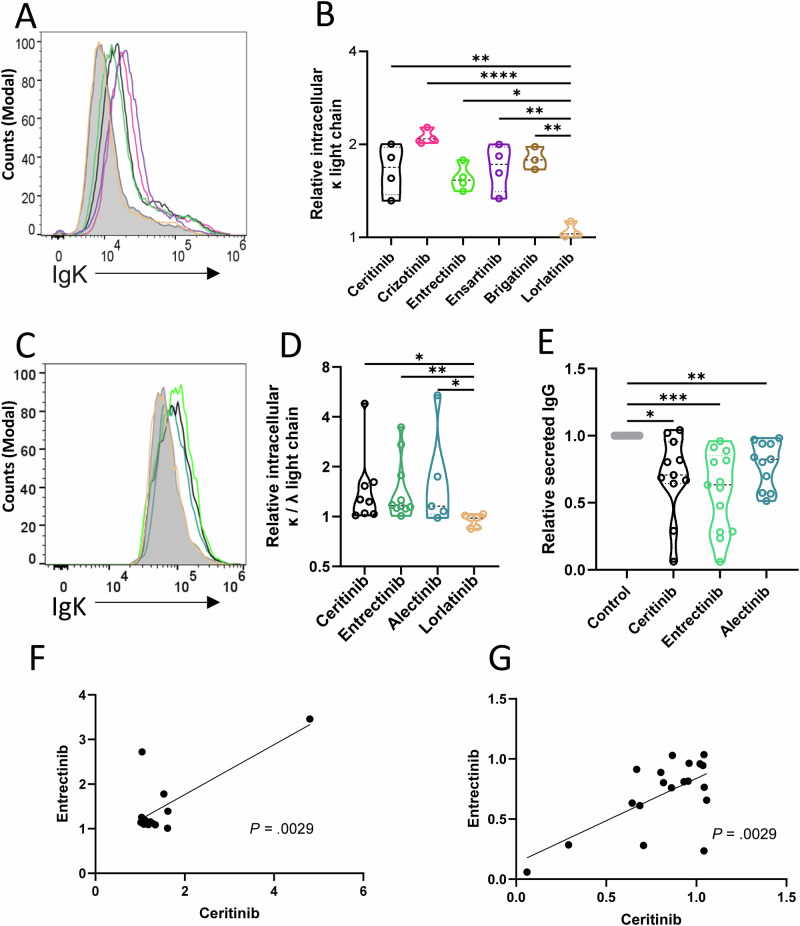
Functional effect of LTK inhibition on Ig secretion inn MM cells. We tested the effect of LTK inhibition on HMCL (**A**, **B**) and primary MM cells (**C**–**G**) in short term culture, in terms of retained intracellular Ig (**A**–**C**) and reduced Ig secretion (**E**) and investigated the covariance of Ig secretion with entrectinib and ceritinib treatment. **A** The URVIN cell line was treated with 5 µM of ceritinib (black), crizotinib (pink), entrectinib (green), ensartinib (purple), brigatinib (brown) and the control lorlatinib (beige) for 18 h or left untreated (DMSO, gray filled histogram). Intracellular staining intensity for κ light chain is shown. **B** Relative expression is correlated to untreated cells. Statistical comparisons to lorlatinib, a low-activity control, is shown (*n* = 4 biological replicates of URVIN, one-way ANOVA). **C** Primary MM cells were treated for 3 h with 5 µM ceritinib (black), entrectinib (green), alectinib (orange) or lorlatinib (beige) or left untreated (gray histogram) for 3 h. **D** The change in intracellular κ or λ light chain (appropriate κ or λ per patient) is shown for primary myeloma samples (ceritinib, *n* = 7, entrectinib *n* = 10, alectinib *n* = 5, lorlatinib *n* = 7). **E** Secreted M-protein from MM cell cultures in assays without drug, after treatment for 3 h (ceritinib, *n* = 11, entrectinib *n* = 14, alectinib *n* = 11). **F** Linear regression analysis of increased light chain stain in MM samples (as in **C**, **D**) after treatment with ceritinib vs entrectinib (*n* = 13). **G** Linear regression of inhibited secretion of M-protein in primary MM cultures (as in **E**) with ceritinib vs entrectinib (*n* = 19). Kruskal–Wallis comparisons with Dunn’s multiple comparisons test in (**D** and **E**) are indicated, *p* < 0.05 (denoted by *), *p* < 0.01 (**) and *p* < 0.001 (***). See also Supplementary Tables S4 and S5 for patient information.

To further define specific functional correlates of LTK inhibition, we tested whether LTK inhibition caused intracellular retention of M-protein and correspondingly reduced secretion ability. We first tested the various ALK-inhibitors on the IgGκ-secreting HMCL URVIN, which was established by us [22]. After 18 h incubation, intracellular flow cytometry showed M-protein retention, detected by intracellular anti-κ light chain staining (Fig. 4A, B) in response to ceritinib, crizotinib, entrectinib, ensartinib, brigatinib and lorlatinib. All of these ALK-inhibitors except lorlatinib (that has a 38x reduced IC50 for LTK [36]) caused an increased mean fluorescence intensity (MFI) of κ light chain staining, (Fig. 4A). Lorlatinib had no effect, with κ light chain staining overlapping with untreated URVIN cells. The intracellular retention of κ was quantified in Fig. 4B, and showed a significant increase for all drugs with activity against LTK compared to lorlatinib (*n* = 4).

The intracellular retention experiments were repeated with freshly isolated CD138^+^ primary MM cells in a short-term 3 h assay to ensure MM cell viability. Even in 3 h assays, we observed increased staining intensity of the appropriate intracellular light chain in cells treated with ceritinib, entrectinib, and alectinib, but not with lorlatinib. An example from one primary patient sample where a shift to the right demonstrates a significant retention of intracellular light chains is shown in Fig. 4C. Quantification of the patient samples (*n* = 5–10) revealed significant M-protein (appropriate light chain) retention when comparing the ALK-inhibitors with lorlatinib (Fig. 4D). With intracellular retention of M-protein, it is expected that there would be a reduced secretion of M-protein from the cells. We therefore measured M-protein in supernatants in further patient samples after 3 h of inhibition, and found a significantly reduced secretion (Fig. 4E, *n* = 11–20).

As the various ALK-inhibitors may differ in off-target effects, we investigated whether the M protein retention in primary MM cells was a common feature that correlated across different drugs. Entrectinib is an ALK, NTREK1-3 and ROS1 inhibitor [37], while ceritinib is an ALK, IGF1R, INSR, TSSK1B and FLT3 inhibitor [38]. We found that both the intracellular retention of M protein (*n* = 12, *p* = 0.0029, Fig. 4F) and the reduced secretion (*n* = 18, *p* = 0.0029, Fig. 4G) showed a linear correlation between these two drugs, strongly suggesting that the observed retention was a direct result of LTK inhibition and not a consequence of different potential off-target effects. MM cells are generally negative for NTREK1-3 (present in 12–30% of patients), ROS-1 (0%), and TSSK1B (0%), but can be positive for IGF1R (43%), INSR (100%), and FLT3 (44%), data from CoMMpass, not shown. These data show that inhibition of LTK results in intracellular retention of M-protein and a significant reduction of antibody secretion from MM cells.

## Discussion

In the current work, we identified LTK as a novel target for the treatment of MM. We demonstrated that inhibiting the biosynthetic branch of proteostasis, regulated by LTK, induces ER stress and ultimately apoptosis in vitro. RNA sequencing data from 767 patients in the CoMMpass study revealed that 83% of patients expressed LTK, while ALK expression was absent. Subsequent qPCR analysis confirmed LTK expression across various stages of MM, including smouldering myeloma, newly diagnosed MM, and relapsed/refractory MM. MM cells expressed over ten times higher LTK levels than healthy donor plasmablasts. Mass spectrometry verified LTK presence at the protein level in all tested samples, including those from relapsed/refractory patients with lower LTK levels. Further analysis revealed the primary isotype of LTK in MM cells as the LTK-202 isoform, which lacks exon 7 and localizes to the ER, as confirmed by co-localization studies in HeLa cells and primary myeloma samples. Functional assays revealed that LTK acts as a regulatory node in the proteostasis network, particularly influencing the unfolded protein response (UPR). In IgM expressing HeLa cells, LTK inhibition increased ER stress markers, particularly under conditions of high secretory load, supporting these predictions. Additionally, MM cells treated with ALK inhibitors, including crizotinib, ceritinib, and alectinib, demonstrated a concentration-dependent reduction in cell viability in concentrations that are in the range (3.1–11.29 µM) measured in serum from patients [39, 40], affecting both proteasome-sensitive and resistant cells. This was also demonstrated in three patients that were sensitive to ceritinib and entrectinib, despite being resistant towards bortezomib. Extending these findings to primary MM cells from patients refractory to proteasome inhibitors (PIs) showed similar results, with crizotinib reducing cell viability across patients with different response statuses to prior treatments. In addition, we demonstrated that in bortezomib sensitive patient cells, there was a significant additive effect of combining bortezomib and ALK-inhibitors. LTK inhibition caused intracellular retention of the M-protein and decreased its secretion, as shown in both HMCLs and primary MM cells. This retention and reduced secretion were observed consistently across treatment with various ALK inhibitors, suggesting that these effects were specific to LTK inhibition rather than off-target activities. Moreover, the correlation between M-protein retention and LTK inhibition across different drugs highlighted the potential of LTK as a therapeutic target in MM. Overall, the data demonstrated that targeting LTK disrupts cell viability by inducing ER-stress and impairing protein secretion, underscoring its therapeutic potential in managing MM.

MM therapy has significantly advanced in the last 20 years, including the introduction of PIs, such as bortezomib, ixazomib, and carfilzomib; immunomodulatory agents (IMIDs), like lenalidomide and pomalidomide; monoclonal antibodies targeting myeloma cell surface antigens (CD38: daratumumab and isatuximab; SLAMF7: elotuzumab); and autologous hematopoietic stem cell transplantation [41, 42]. Overall survival for newly diagnosed MM has dramatically improved from about 30 months before the year 2000 [43] to more than 120 months with standard first-line therapy comprising lenalidomide, bortezomib, and dexamethasone induction therapy [44]. Another common triplet therapy is daratumumab, lenalidomide, and dexamethasone (DRd) [41, 42]. Almost all patients with MM eventually relapse, and median progression free survival (PFS) and overall survival (OS) in patients with relapsed MM refractory to lenalidomide and bortezomib is poor, with median times of 5 months and 9 months, respectively [45].

Relapsed patients are mostly treated with triple therapies, but triple-refractory MM patients (refractory to a PI, an immunomodulatory drug (IMID) and an anti-CD38 mAb) and penta-refractory MM patients (refractory to two PIs, two IMIDs and an anti-CD38 mAb) are not uncommon. Thus, new agents are required for such multi-refractory patients. Selinexor, a selective inhibitor of nuclear export (SINE), has shown promise for these patients [46]. This drug covalently binds to the cargo-binding groove of exportin-1 (XPO1), interfering with protein trafficking from the nucleus to the cytosol. XPO1 inhibition causes the accumulation of tumor suppressor proteins such as p53, p21, and p27 in the nucleus, reactivating tumor suppressive pathways, inducing cell cycle arrest, and promoting pro-apoptotic pathways leading to apoptosis in cancer cells [47].

Our findings that LTK is expressed in MM cells, combined with the observation that this kinase was activated by secretory flux, make LTK a promising drug target for myeloma therapy. Moreover, the current demonstration that LTK inhibition remains effective in PI-refractory patients suggests that LTK inhibition could play a role in the treatment of MM patients refractory to one or more PIs. Furthermore, as LTK inhibition targets the biosynthetic part of the proteostasis network and the secretory pathway, it could add to PI inhibition of the degradation part of the proteostasis network and be part of combinational therapies. SINE and PI are already a potent combinational therapy [48], and a third potential synergy could involve SINE with the two other parts of the proteostasis network, targeting the biosynthetic part and secretory pathway (LTK) and protein degradation (proteasome).

LTK inhibition is of special interest in MM as even in end-stage MM a hypersecretory phenotype of MM cells is maintained in most patients, as indicated by persistently increased M-protein levels. Early estimates suggested that individual MM cells secrete up to 85,000 molecules of IgG per minute [3]. Current results demonstrate that MM cells likely depend on proper LTK function, the first discovered ER-resident receptor tyrosine kinase that regulates export from the ER [14, 49]. The ER is a major site for proteostasis, but little was known about the adaptation to higher secretory load of appropriately folded protein and the regulation at ER entry sites [11–13]. We showed an upregulation of LTK expression in HeLa cells as an adaptation to IgM secretion, and that such cells, but not non-transfected cells, became sensitive to LTK inhibition. Further, LTK correlated with IgG1 expression in IgG1^+^ MM and that short-term inhibition resulted in M-protein retention and apoptosis of MM cells regardless of prior therapy.

Besides having relevance for plasma cell malignancies, our results may also be of interest for other diseases featuring hypersecretion. Non-malignant plasma cells that also secrete high levels of Ig are relevant targets, especially in autoantibody-driven autoimmune disorders. Interestingly, a gain-of-function mutation in LTK was identified in the LTK kinase domain in lupus-prone mice and in patients with systemic lupus erythematosus [50]. In light of the current results, increased LTK function could potentially play a role in B cell differentiation and the generation of plasma blasts as well as long-lived plasma cells.

Besides LTK inhibition, the ALK-inhibitors, tested here, may have potential beneficial synergistic effects in MM due to the inhibition of various kinases by each particular drug. For example, ceritinib has activity on IGF1R and INSR, both of which may impact metabolically active MM cells. Inhibition of MET, the most MM-relevant non-ALK/LTK target for crizotinib, would not induce ER-stress [51] nor cause M protein retention, but may play a role in inhibiting MM responses to HGF including reduced proliferation, and microenvironment effects [52]. However, inhibition of other target kinases could not explain the findings of M-protein retention within the 3-hour scope of our assays. In this regard, the finding of a linear correlation with the effect of ceritinib versus entrectinib (ALK, NTRK1-3) strongly argues against other targets than LTK.

The results suggest that MM cells are particularly vulnerable targets of LTK inhibition and suggest that potential synergies may be favorable both in combination with existing therapies (PIs and SINE) and may provide additional MM-relevant inhibition for repurposed ALK inhibitors.

## Supplementary information

Supplemental material

## Data Availability

All material described in the manuscript, including all relevant raw data will be freely available to any researcher wishing to use them for non-commercial purposes, without breaching participant confidentiality. Access to patient sensitive data is restricted (REC#2016/947 and 2012/174).
