# Supplementary material for: Targeting proteostasis in multiple myeloma through inhibition of LTK
Source: Leukemia. 2025 Jul 9;39(9):2237–45. doi: 10.1038/s41375-025-02682-8 (PMC12380618; doi:10.1038/s41375-025-02682-8)
Supplement: Supplementary file 1 — Supplemental material [file 41375_2025_2682_MOESM1_ESM.pdf]

Supplementary Information, Våtsveen et al, "Targeting Proteostasis in Multiple Myeloma through Inhibition of LTK"

Inventory

Supplementary Methods

Drug treatment and cell viability assay

RNA isolation and RT-PCR

DNA construct

CRISPR

Immunoblotting

Mass spectrometry

Immunofluorescence

Supplementary Figure Legends

Supplementary Figure S1. LTK in MM, copy number, M protein, common MM mutations, and intracellular localization

Supplementary Figure S2. Induction of ER stress in HMCLs and combinatorial treatment with bortezomib.

Supplementary Figure S3. Viability curves for 6 patients shown in Figure 4 and drug effects on HMCL

Supplementary Figure S4. Mutations and gene expression in HMCL.

Supplementary Tables

Table S1. Patient information related to Figure 3B-E

Table S2. Patient information related to Figure 3H and 3I

Table S3. Patient information related to Figure 3H, I and Supplementary Figure S2A

Table S4. Patient characteristics related to Figure 4 and Supplementary Figure S5

Supplementary Methods

Drug treatment and cell viability assay

$CD138^+$ MM cells (5000-10,000 cells/well) from activation assays were tested in 384-well plates against ALK-inhibitors at a 0.01-10 µM concentration range as previously described (17). Plates were dispensed using an Echo acoustic dispenser, LabCyte Inc., San Jose, CA, USA) and incubated in a humidified environment at 37°C with 5% CO_2. Cell viability was determined after 72h using the CellTiterGlo ATP assay (Promega, Madison, WI, USA) following the manufacturer's instructions, with luminescence measured on Envision Xcite plate reader (Perkin Elmer, Shelton, CT, USA). Relative cell viability was calculated by normalizing luminescence values to negative controls (0.1% DMSO) and positive control (100 µM BzCl) followed by curve-fitting. Drug sensitivity score (DSS) was calculated as previously described (18). Bortezomib 10 – 0.04 nM (Selleckhem) was used for combinatorial treatment with entrectinib (0.04-10 µM) and ceritinib (0.04-5 µM) in 72h assays using CellTiterGlo. Data was analyzed using Combenefit (Online tool to analyze drug combinations GY Di Virioli et al 2012). The viability of L363 and L363-BTZ cells was determined 24 h post-treatment using the Cell Counting Kit-8 (CCK-8; MedChemExpress, NJ, USA) according to the manufacturer's instructions.

RNA isolation and RT-PCR

Total RNA was isolated from cell lines using TRIzol (Ambion/Thermo Fisher Scientific, MA, USA) and Direct-zol RNA MiniPrep (Zymo Research, CA, USA) or RNeasy kit (Qiagen). 500-1000 ng of total RNA was reverse transcribed using the High Capacity cDNA Reverse Transcription kit (Applied Biosystems/Thermo Fisher Scientific, MA, USA) according to manufacturer's recommendations. Subsequently, 10 ng of cDNA was used into qPCR reactions with 2x PowerUp SYBR Green Master Mix (Applied Biosystems/Thermo Fisher Scientific, MA, USA) and the following primers: spliced, unspliced and total XBP1, ATF4 and GAPDH as a housekeeping gene using a QuantStudio5 Real-Time PCR system (Applied Biosystems/Thermo Fisher Scientific, MA, USA) or 2x Applied Biosystems TaqMan Universal PCR Master Mix with ALK Hs00608284_m1, LTK Hs01587782_g1 and GAPDH Hs09999995_m1 was used for the HMCL and the primary patient samples on a 7500 Real Time PCR System (Applied Biosystem).

DNA construct

The pcDNA-LTKΔexon7 construct was generated by site-directed PCR mutagenesis using a full-length construct as template with the following primers:

5'-AGGAGGAGCGGCCGGGGGAGATGCAAGTGAGACCGAC-3',

5'-CCCGGCCGCTCCTCCTCTTCCGCCACTACCGGGTGC-3'.

GFP-LTKΔexon7 was generated using NEB HiFi Assembly. The backbone fragment encoding LTK was amplified by PCR from the template pcDNA-LTKΔexon7 with the following primers:

5'-GCTGTACAAGGGAGGTGGTGGGTCCTCTAGCCCGGGGTCCCAG-3',

-GGATCCAAGGTACCTAGCTAGCC-3'; The fragment encoding mGFP and the prolactin signal sequence was amplified by PCR from a GFP-ERGIC-53 construct (28)

using primers: 5'-TAGCTAGGTACCTTGGATCCATGGACAGCAAAGGTTCG-3',

5'-CCGGGCTAGAGGACCCACCACCTCCCTTGTACAGCTCGTCCATG-3'.

CRISPR

SgRNA sequences targeting exons 2, 4 and 5 of human *LTK* (Ltk_e2: 5'-GTGGCTCCAAGATACTAGGCG-3', Ltk_e4: 5'- GCGCCAAGAACCACCTGTCGC

Ltk_e5: 5'-GCGGGGAGTCTCGAGCCGTTG-3') were designed using the web-based CHOPCHOP platform, cloned into the PX458 expression vector (Addgene #48138, provided by Dr. Feng Zhang through the Addgene repository), and transiently expressed in RPMI-8226, JJN-3, URVIN and FOLE using the Neon NxT electroporation system (ThermoFischer Scientific). After 24h, GFP-expressing cells were sorted by fluorescence-activated cell sorting. Following recovery, individual clones were obtained by limiting dilution. Genomic DNA was isolated from individual clones, and screened by Sanger sequencing for non-sense mutations in LTK, and qPCR (described above) was used to verify if LTK expression was interrupted.

Immunoblotting

The day after transfection HeLa cells with LTKΔexon7 or control constructs were treated with 1 μM of crizotinib, ceritinib or DMSO for 30 min before lysis in RIPA buffer, containing Pierce Protease and Phosphatase Inhibitor (A32961, ThermoFisher)). Lysates were separated by SDS-PAGE on a 6% polyacrylamide gel and transferred to nitrocellulose membranes. Membranes were blocked with 5% w/v nonfat dry milk in PBS with 0.1% Tween-20 and incubated with primary antibodies probed with rabbit anti-LTK mAb (1:1000 , ab129155, Abcam), rabbit anti-pLTK mAb (1:1000, CellSignaling), rabbit anti-XBP1 (1:1000, poly6195, Bio Legend), mouse anti-beta actin (C4, sc47778, Santa Cruz), mouse anti-vinculin mAb (1:2500, VIN-11-5, SAB4200729, Sigma-Aldrich), followed by HRP-labeled goat secondary antibodies (111-035-003, 1115-035-004, Jackson ImmunoResearch) Visualization was done using ECL (BioRad) on a ChemiDoc imaging system (BioRad).

Mass spectrometry

Protein lysates were prepared using RIPA buffer from 5-10 x10^6 primary myeloma cells or HMCLs and incubated with 2.5 μg of anti-LTK, Rabbit, Sigma-Aldrich, HPA059545 per 100 μl lysate. Pierce Protein A/G magnetic agarose beads were used according to manufacturer's instructions (ThermoFischer Scientific). Immunoprecipitated proteins were reduced, alkylated and digested with trypsin directly on the beads. The resulting peptides were purified using home-made C18 Stage-tips and were analyzed using LC-MS/MS (EVOSEP coupled to QExactiveHF via an EasySpay nano-electrospray ion source, Thermo Scientific). Protein identification was done using MaxQuant software (version 2.0.1.0), by database search against human sequences from UniProt. Mass spectrometry-based proteomic analyses were performed by the Proteomics Core Facility, Department of Immunology, University of Oslo/Oslo University Hospital.

Immunofluorescence

HeLa cells grown on glass coverslips were transfected with a construct encoding GFP-LTKΔexon7 using a 1:3 (w/w) mixture of DNA to polyethylenimine. After washing with PBS, cells were permeabilized with 0.2% Triton-X-100 in PBS for 5 min and blocked with 5% BSA in PBS containing 0.1% Tween-20 for 30 min. Samples were incubated with Rabbit anti-CLIMP63 (CKAP4) pAb (1:500, a gift from Hans-Peter Hauri) in blocking buffer for 2 h at RT, followed by goat anti-rabbit AlexaFluor®-568 labeled secondary antibody (A11011, ThermoFisher) in blocking buffer for 1 h at RT. Coverslips were mounted in polyvinyl alcohol

mounting medium with DABCO (10891, Sigma-Aldrich) and analyzed on a Nikon Eclipse Ti2 spinning disc confocal microscope (Crest X-Light v2) equipped with a Nikon Apochromat TIRF 60 x/1.49 Oil objective, SPECTRA Light Engine (Lumencor) and a Prime BSI sCMOS camera (Teledyne Photometrics).

Supplementary Figures

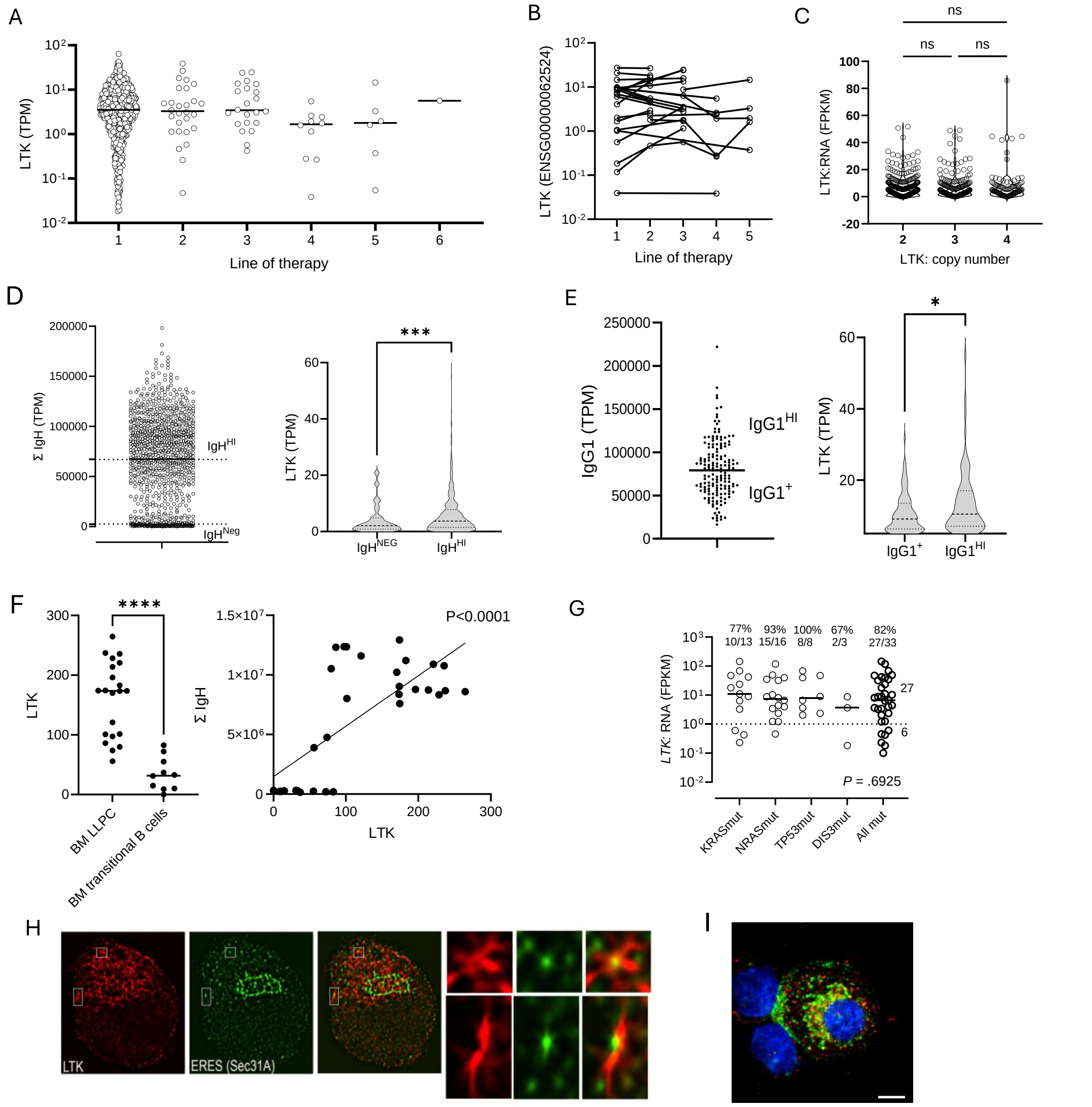

Supplementary Figure S1. LTK in MM, copy number, M protein, common MM mutations, and intracellular localization

A) LTK expression (ENSG00000062524) vs lines of therapy (CoMMpass, IA-17), all patients (left) and **B)** paired data (right). No significant LTK variation with lines of therapy left, one-way ANOVA, P=0.389; right, Mixed effects analysis, paired data P=0.3554). **C)** LTK expression (ENSG00000062524) in MM cells in (CoMMpass, IA-13), LTK copy number vs LTK RNA expression is shown. We found no significant change in LTK expression in myeloma patient cells vs. copy number changes, one-way ANOVA P=0.487. **D)** . Left: Expression of sum of IgH chain transcripts (∑ IgH TPM) of IgG1, IgG2, IgG3, IgG4, IgA1, IgA2; ENSG00000211896, ENSG00000211893, ENSG00000211897, ENSG00000211892, ENSG00000211895, ENSG00000211890; CoMMpass, IA-17) in MM cells, identifying MM cells that have lost IgH chain expression (IgH^{Neg}) and patients with above mean IgH expression (IgH^{HI}), mean is shown (dotted line). Right: Expression of LTK in MM cells without

IgH secretory burden (IgH^{NEG}) vs. patients with MM expression IgH^{HI}, Mann-Whitney, P=0.0009. **E)** LTK expression related to secretion of IgG1 Heavy chain, left: IgG1 expression (ENSG00000211896) is shown, identifying patients with above average IgG1 expression ($IgG1^{HI}$) and patients with lower than average IgG1 expression ($IgG1^{+}$). Right: LTK expression is shown in the two groups, Mann-Whitney, P=0.0277. **F)** Bulk mRNA sequencing, expression of LTK in bone marrow B cells. Left: LTK expression in long lived plasma cells (LLPC, $CD19^{+/-}$) vs. early mature B cells (Transitional B cells) in SLE patients and healthy controls. Mann-Whitney, P<0.0001. Right: LTK vs sum of IgH chain transcripts (normalized counts ∑ IgH of IgM, IgD, IgG1, IgG2, IgG3, IgG4, IgA1, IgA2, IgE), simple linear regression is shown. Data was kindly provided by Dr Jennifer Barnas[1]. **G)** LTK expression in MM patients with common mutations (CoMMpass, IA-13), no significant differences in the groups were seen, one-way ANOVA, P=0.6925. H) LTK localization and interaction with cargo receptors. RPMI-8862 HMCL were fixed and stained for endogenous LTK, with LTK antibody (1:100, Rabit, Abcepta) in red and Sec31A (1:100, Mouse, Clone: 32, BD) in green. Cells were imaged using SRRF stream superresolution miccroscopy. **I)** Image of a myeloma cell LTK (red), sec31A (green) from a newly diagnosed IgG Lambda secreting myeloma patient. The cells were attached by cytospinn, air dired, and stained with LTK (1:100, Rabbit, Sigma-Aldrich, HPA059545) , and Sec31A (1:100, Mouse , Clone: 32, BD) in green , Secondary antibodies (1;500) Alexa Fluor 488 Donkey anti-Mouse, Alexa Fluor 555 Donkey anti-Rabbit (Thermo Fisher), scarebar corresponds to 5 microns, 100 X UPlanFL objective, Olympus FV1000 confocal microscope.

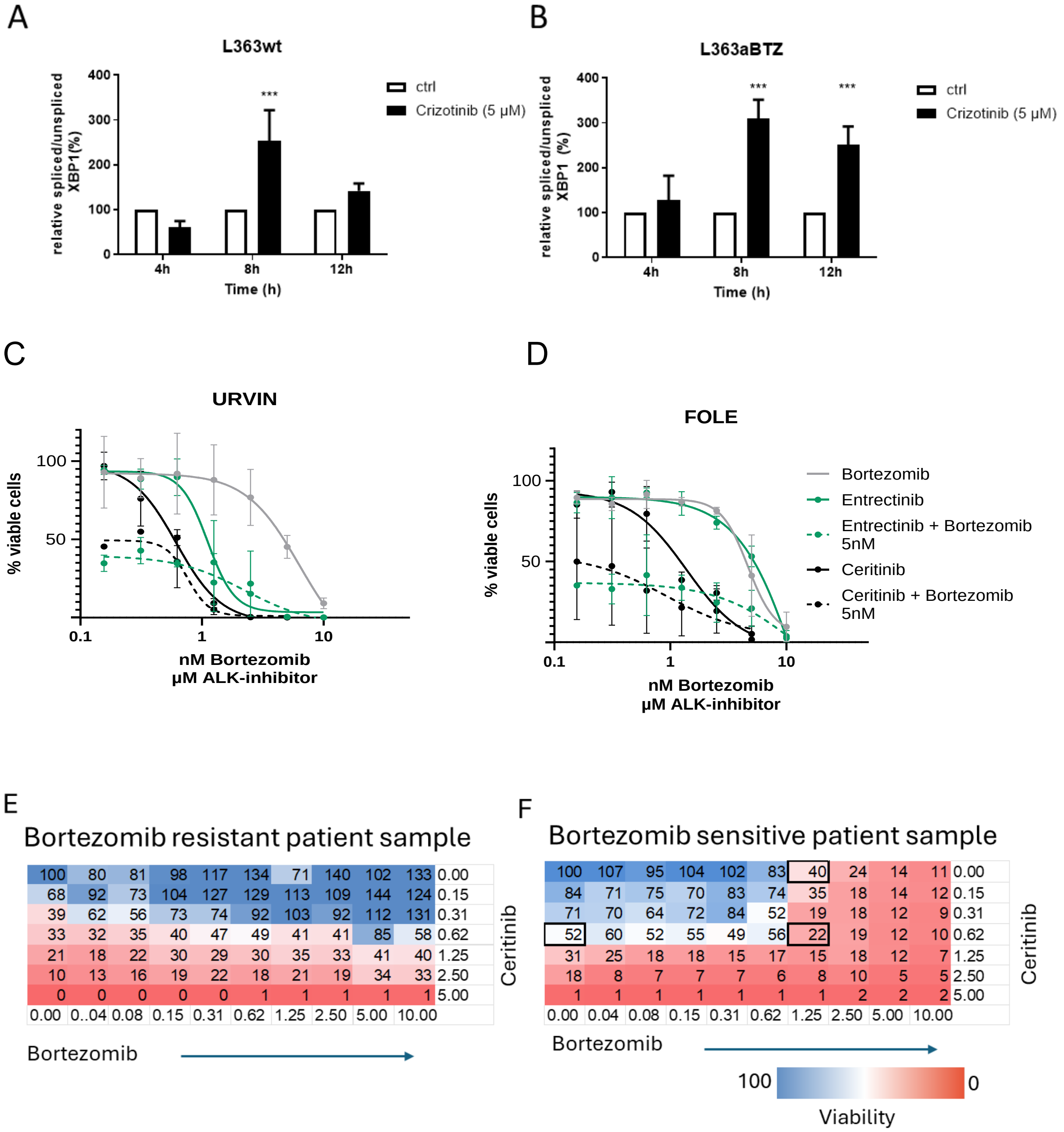

Supplementary Figure S2. Induction of ER stress in HMCLs and combinatorial treatment with bortezomib

HMCL L363, wild type **A)** and bortezomib-resistant L363-BTZ cells **B)** were treated with crizotinib (5 μM) and splicing of XBP1 (ER stress) was measured. Time course (4, 8 and 12h) of crizotinib treatment (black bars) or control (open bars). The ΔCt method is used for fold change calculation and control is set as 100 % for calculations of change in the spliced/unspliced XBP1. Two-way ANOVA test is shown. **C** and **D)** HMCLs URVIN and KJON were treated with a combination of bortezomib (10 nM-0.04 nM) and ceritinib (5 μM- 0.16 μM) or entrectinib (10 μM – 0.16 μM) for 48 h. At ≈5 nM bortezomib, 50% of the treated cells were viable; we show this concentration in combination with ceritinib and entrectinib (dotted lines). **E)** Representative heatmap of a bortezomib resistant patient showing data from all

tested drug combinations, viability (0-100) is shown for each drug combination, or without drug (upper left, indexed to 100). Data corresponds to selected data shown in Fig 3C. **F)** Representative heatmap of a bortezomib sensitive patient, as in E with values picked for Fig 3D highlighted. Viability is shown in a blue-white-red color scale (as indicated).

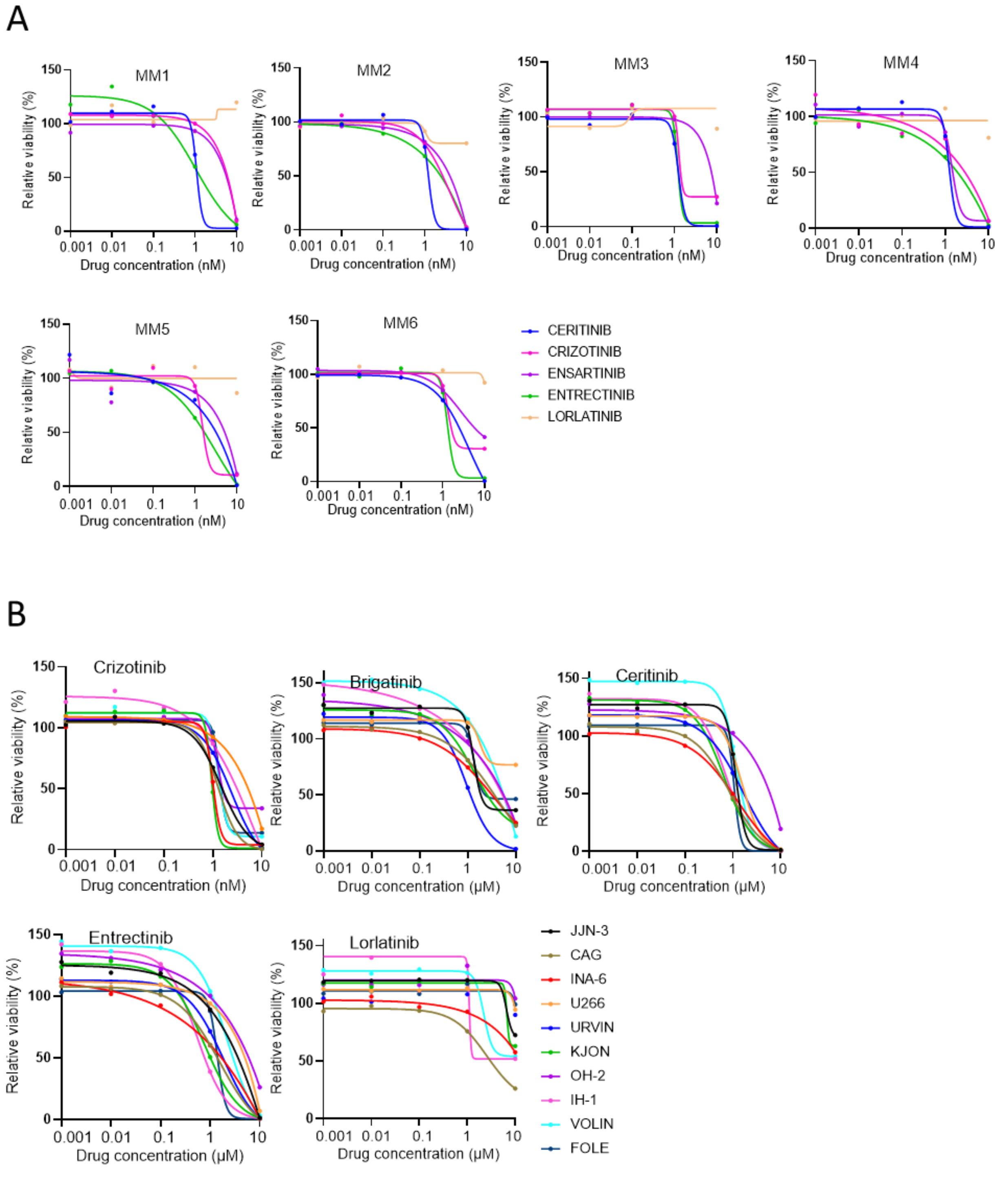

Supplementary Figure S3. Viability curves for 6 patients shown in Figure 4 and drug effects on HMCLA) Viability curves of CD138^{+} MM cells are shown for each individual patient from Figure 4F. Patient cytogenetics and treatment lines are shown in Supplementary Table S2. **B)** HMCLs viability curves are shown for the different inhibitors. All data were obtained with the CellTiterGlo assay, 72 h incubation.

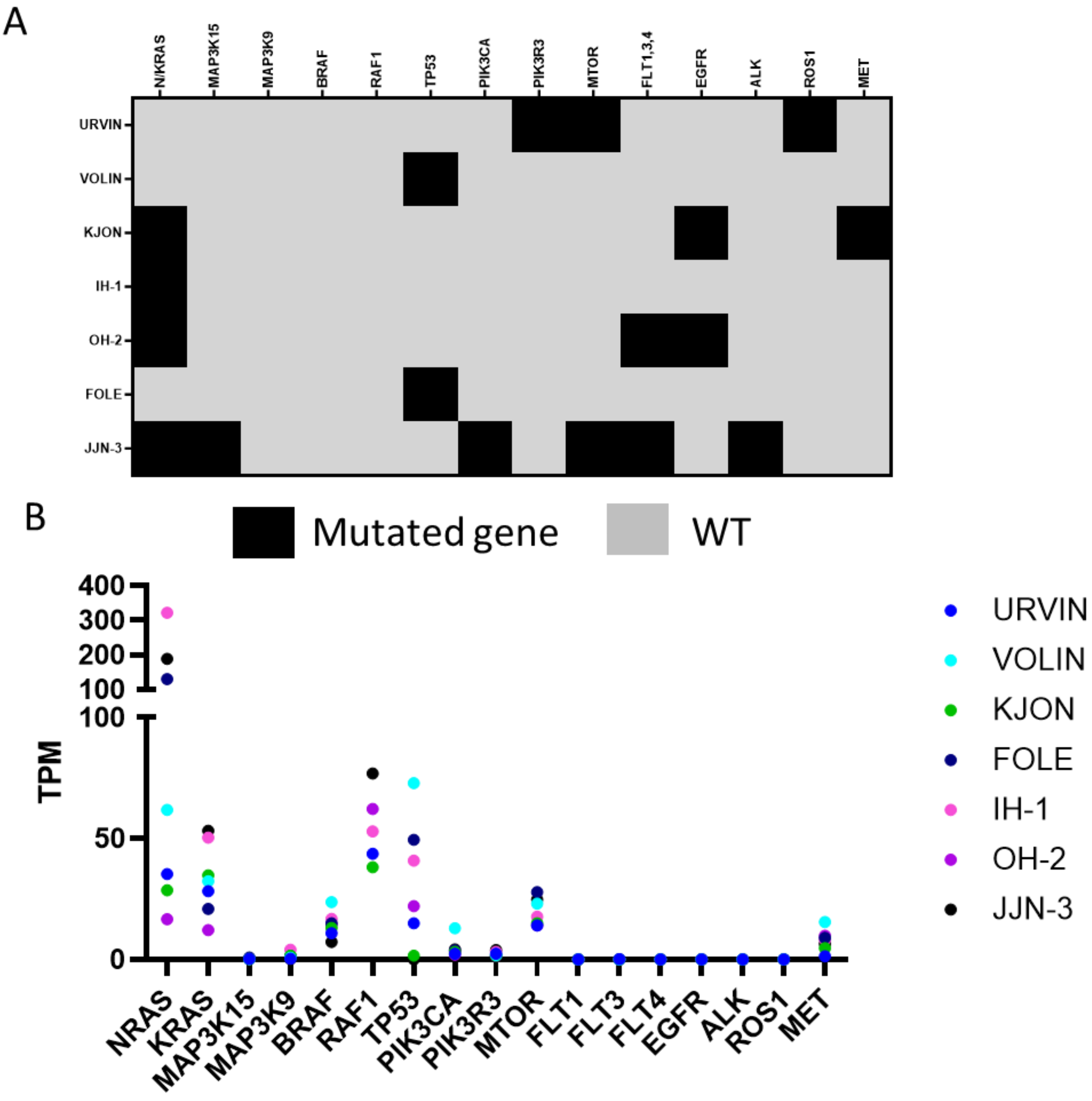

Supplementary Figure S4. **Mutations and gene expression in HMCL**

A) Mutations in frequently mutated genes in cancer in the HMCL used in this study. Black boxes indicate mutations in the genes as indicated in the top row. Unmutated (WT) genes are shown in gray. **B)** Gene expression in HMCL. RNA transcripts (TPM, Transcript per million) of the same genes from the HMCL, as indicated. Further data (not presented in this figure) include person identifiable data and cannot be openly shared according to Norwegian legislation. Data can only be shared on a collaborative basis based on approval from the Regional Ethics Committee. Requests to access the datasets should be directed to the corresponding author. Processed data from RNA sequencing can be found in GEO under accession number GSE250236.

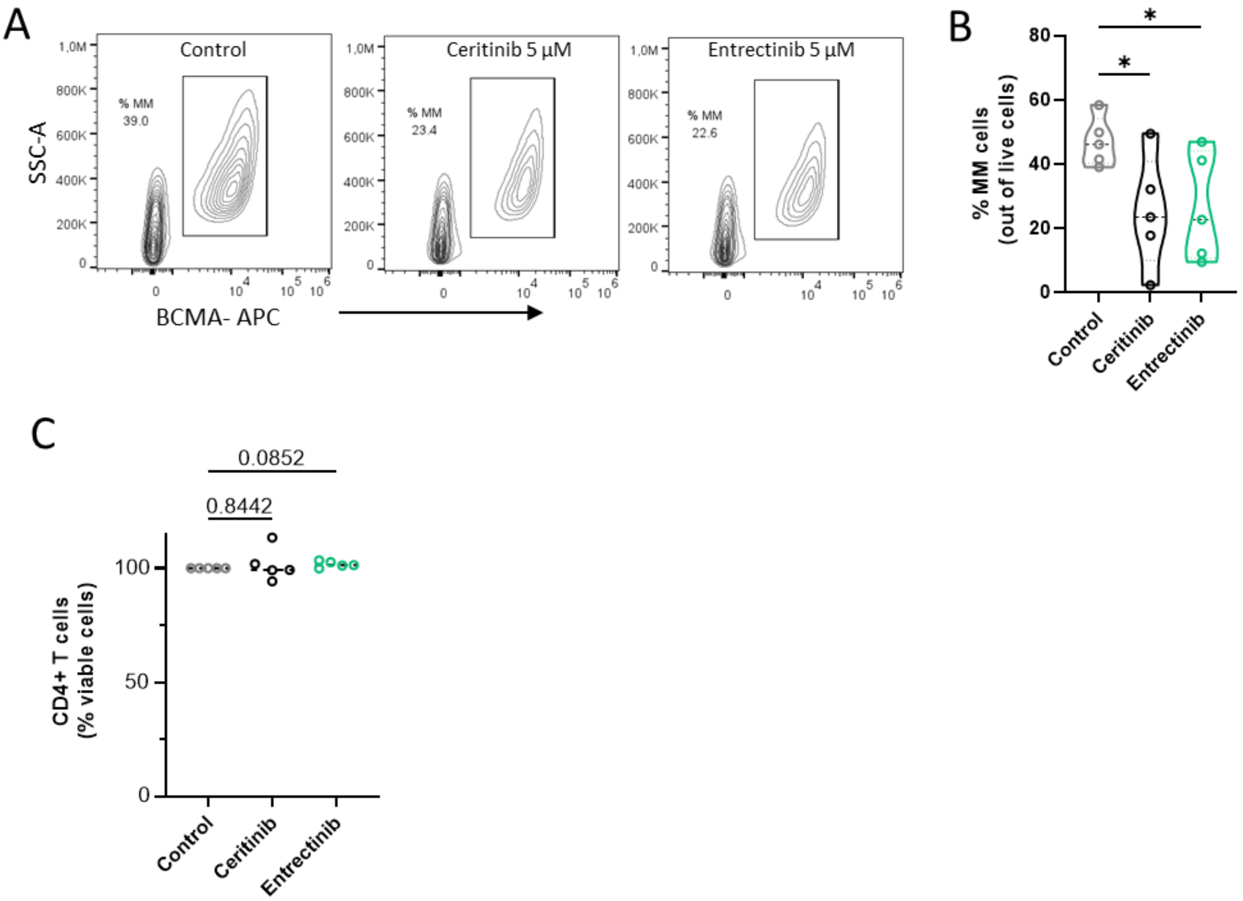

Supplemental Figure S5. Ceritinib and entrectinib significantly reduces the percentage of myeloma cells in BMMCs from freshly harvested bone marrow.

BMMC were cultured with ceritinib and entrectinib (5 µM) for 48 h. Myeloma cells were identified as BCMA$^+$ by flow cytometry and the percentage of myeloma cells out of total live cells was measured. **A)** Flow cytometry contour plots (BCMA vs. side scatter) showing gated BCMA$^+$MM cells from a representative patient, with DMSO (left), ceritinib (middle) and entrectinib (right). **B)** Percentage MM cells in BMMC cultures from (n = 5) patients treated with DMSO, ceritinib or entrectinib. One-way ANOVA p<0.05 (denoted by *) is shown. **C)** Relative numbers of viable CD4$^+$ T cells in the BMMC in untreated samples (indexed to 1) compared with ceritinib and entrectinib (5 µM), one-way ANOVA (p = .8442 and .0852 respectively).

Table S1. Patient information related to Figure 3B-D

Patient	Stage	M-protein	Lines of therapy
**MM-I**	Relapse	IgG L	1) Cyclophosphamide + Borteszomib + Dexamethasone + ASCT<br>2) Lenalidomide + Dexamethasone<br>3) Targovax
**MM-II**	Relapse	IgA L	1) Bortezomib + Lenalidomide + Dexamethasone<br>2) Daratumumab + Bortezomib + Dexamethasone<br>3) Lenalidomide + Dexamethasone<br>4) Targovax
**MM-III**	First Line	IgA K	
**MM-IV**	Relapse	Lambda	1) Ixasomib + Lenalidomide + Dexamethasone<br>2) Ixasomib + Daratumumab + Dexamethasone
**MM-V**	Relapse	IgG K	1) Bortezomib + Lenalidomide + Dexamethasone
**MM-VI**	First Line	IgG K	
**MM-VII**	Relapse	Kappa	1) Cyclophosphamide + Borteszomib + Dexamethasone<br>2) Bortezomib + thalidomide + Dexamethasone<br>3) Cyclophosphamide + Lenalidomide + Dexamethasone + ASCT<br>4) Doxorubisine + Melphalan + Dexamethasone + ASCT<br>5) Mezigdomide + Carfilzomib + Dexamethasone
**MM-VIII**	First Line	IgA K	
**MM-IX**	First Line	IgG K	

ASCT: autologous stem cell transplant

Table S2. Patient information related to Figure 3E and 3F

Patient	% PC	M-protein	ISS	FISH	Diagnosis at sampling	Line. Therapy
**MM-A**	40 %	IgA 22 g/L	2	No information	Relapsed	1. Melphalan+Prednisolone+Bortezomib<br>2. Lenalidomide+Dexamethasone
**MM-B**	65 %	IgG51,5g/L	2	Negative	Relapsed	1. Cyclophosphamide+Bortezomib+Dexamethasone +ASCT<br>2. Carfilzomib+Cycclofosfamid+Dexamethasone +ASCT+Carfilzomib maint<br>3. Lenalidomide+Dexamethasone
**MM-C**	20 %	IgG<br>K 11800 mg/L<br>L 15,9 mg/L	3	add(1q21), add(1p32), add(11q13), add(6p21), add(20q12), del17	Relapsed	1. Bortezomib+Dexamethasone<br>2. Cyclophosphamide+Thalidomid+Dexamethasone +ASCT<br>3. Bortezomib+Lenalidomide+Dexamethasone +ASCT+Lenalidomide<br>4. Bortezomib+Lenalidomide+Dexamethasone
**MM-D**	60 %	IgG<br>62 g/L<br>L 1110 mg/L	2	del(17q13), del13q, del(20q12)	Relapsed<br>Relapsed	1. Bortezomib+Lenalidomide+Dexamethasone<br>2. Daratumumab+Cyclophosphamide +Dexamethasone<br>3. Daratumumab+Bortezomib+Dexamethasone
**MM-E**	-	-	-	Negative	Relapsed	1. Melfalan+Prednisolon+Thalidomide<br>2. Cyclophosphamide+Bortezomib +Dexamethasone+ASCT+Lenalidomide<br>3. Panobinostat+Bortezomib+Dexamethasone
**MM-F**	44 %	IgG<br>6,8 g/L	1	Negative	Relapsed	1. CyDex+ASCT+Bortezomib<br>2. Cyclophosphamide+Bortezomib +Dexamethasone+ASCT<br>3. Lenalidomide+Dexamethasone
**MM-G**	40 %	IgA<br>38 g/L<br>K 224 mg/L<br>L 8,8	2	del(13q14), del(16q23), del(20q12)	Newly diagnosed	First line
**MM-H**	15 %	IgG 38,8 g/L<br>K 2130 mg/L<br>L 17 mg/L	1	add(1q21), t(11;14)(q13q32), del13q, add(4p16)	Relapsed	1. Cyclophosphamide+Bortezomib +Dexamethasone+ASCT<br>2. Lenalidomide- Dexamethasone<br>3. Bortezomib+Lenalidomide +Dexamethasone
**MM-I**		IgG 5,3 g/L<br>L 27 mg/L<br>K 13 g/L	1	add(11q13), add(6p21), add(20q12)	Relapsed	1. Melphalan+Prednisolone+Bortezomib
**MM-J**	15 %	IgA 24,2 g/L<br>K 21 mg/L<br>L 7 mg/L	1	add (11q13)	Newly diagnosed	First line
**MM-K**	15 %	IgA 15,2 g/L<br>K 1470 mg/L<br>L 10	2	add(11q13), add(6p21), add(16q23), add(1q21)	Newly diagnosed	First line
**MM-L**	75 %	IgG 65 g/L	1	add (1q21), del (17p13), del13q, del(14q32)	Relapsed	1. Cyclophosphamide+Bortezomib +Dexamethasone+ASCT

				Del(4p16), del(16q23), add (6p21)		2. Melfalan+Prednisolon+Bortezomib<br>3. Bortezomib+Lenalidomide+ Dexamethasone+ASCT+Lenalidomide maint
**MM-M**	16 %	IgG 34 g/L<br>k 106 mg/L<br>L 53,3 mg/L	1	t(4;14), add(1p1q)	Relapsed	1. ASCT+Bortezomib+Lenalidomide +Dexamethasone<br>2. ASCT+Bendamustine+Melphalan +Pomalidomide+Dexamethasone<br>3. Panobinostat+Bortezomib +Dexamethasone<br>4. Daratumumab+Carfilzomib +Dexamethasone
**MM-N**	25 %	617 mg/L<br>L 6 mg/L		add(1q21), add (1p32), t(11;14)(q13;q32), del 13q, add (4p16), add(6p21), add(16q23), add (20q12)+tetrasomi 1	Newly diagnosed	First line
**MM-O**	30 %	IgG 38,8<br>K 2130<br>L 17	1	add(1q21), add (11q13), add(6p21)	Relapsed	1. ASCT+Cyclophosphamide +Dexamethasone+Bortezomib<br>2. ASCT+Lenalidomide+Dexamethasone<br>4. Melfalan+Prednisolon+Bortezomib
**MM-P**	-	IgG 20 g/L	-	t(11;14)	Relapsed	1. Cyklofosfamid+Dexamethasone+ASCT<br>2. ASCT+Veldex+CyklofosfamidX6<br>3. Melphalan+Prednisolon+Bortezomib<br>4. Lenalidomide+Dexamethasone<br>5. Melfalan+Prednisolon+Bortezomib<br>6. Lenalidomide<br>7. Daratumumab+Dexamethasone +Bortezomib<br>8. 5x carfilzomib+Dexamethasone +5xCarfilzomib+Dexamethasone<br>9. Bendamustin+Pomalidomide +Dexamethasone
**MM-Q**	**-**	-	-	add(14q23), add (11q13)	Relapsed	1. Vincristine+Adriamycin +Dexamethahsone+ASCT +Interferon<br>2. Melphalan+Prednisolone+Bortezomib<br>3. 8XLenalidomide+Bortezomib +Dexamethasone
**MM-R**	10 %	-	1	add(11q13), del(16q23), del17p, del13q,	Relapsed	1. Bortezomib+Thalidomid +Dexamethasone+ASCT<br>2. 2XBortezomib+Lenalidomide +Dexamethasone+ASCT<br>3. 4XBortezomib+Lenalidomide +Dexamethasone,<br>4. ASCT+Lenalidomide+Dexamethasone
**MM-S**	60 %	-	4	del1p add1q, del17, t(14;16) add17t(14;16)(q32;q;23), add (4p16), add (6p21), add(11q13), add (20q12)	Newly diagnosed	First line
**MM-T**	20 %	-	2	add (14p16), add(11q13)	Newly diagnosed	First line

**MM-U**	30 %	-	1	del1p, add1q, add(11q13), t(14;20)	Relapsed	1. Cyklofosfamid+Dexamethasone +ASCT<br>2. Cyklofosfamid+Bortezomib +Dexamethasone<br>3. Bortezomib+Dexamethasone<br>4. Lenalidomide+Dexamethasone<br>5. Pomalidomid+Dexamethasone<br>6. Cobimetinib+Venetoclax<br>7. Daratumumab+Bortezomib +Dexamethasone
**MM-V**	85 %	-	1	add (1q21), t(11;14)(q13q32)	Relapsed	1. Bortezomib+Cyklofosfamid +Dexamethasone+Melphalan+ASCT<br>2. Bortezomib+Lenalidomide +Dexamethasone+Lenalidomide +Dexamethasone<br>3. Melflufen+Dexamethasone<br>4. Daratumumab+Bortezomib +Dexamethasone<br>5. Daratumumab+Carfilzomib +Dexamethasone

Table S3. Patient information related to Figure 3I, J and Supplementary Figure S2A

Patient	Cytogenetic profile	M-protein	Lines of therapy
**MM1**	t(4;14),<br>gain1q21,<br>del 13q14	Kappa	5) cyclophosphamide+bortezomib+dexamethasone<br>6) lenalidomide+dexamethasone+ASCT<br>7) carfilzomib+dexamethasone<br>8) lenalidomide+dexamethasone<br>9) daratumumab+bortezomib+dexamethasone+ASCT<br>2)
**MM2**	t(14;20),<br>gain1q21,<br>del1p32,<br>gain11q13,<br>gain14q32	Kappa	5) cyclophosphamide+dexamethasone+ASCT<br>6) cyclophosphamide+bortezomib+dexamethasone<br>7) bortezomib+dexamethasone<br>8) lenalidomide+dexamethasone<br>9) pomalidomide+dexamethasone<br>10) cobimetinib+venetoclax<br>11) daratumumab+bortezomib+dexamethasone
**MM3**	t(11;14)	Kappa	1) cyclophosphamide+bortezomib+dexamethasone<br>2) NA<br>3) pomalidomide+dexamethasone<br>4) NA<br>5) cyclophosphamide+bortezomib+dexamethasone
**MM4**	None detected	Kappa	3) cyclophosphamide+bortezomib+dexamethasone +lenalidomide+ASCT<br>4) bortezomib+thalidomide+dexamethasone<br>5) panobinostat+bortezomib+dexamethasone<br>6) carfilzomib+dexamethasone<br>7) NA<br>8) NA<br>9) Isatuximab+lenalidomide+dexamethasone
**MM5**	NA	IgG 29 g/L	2) melphalan+prednisolon+bortezomib<br>3) lenalidomide+dexamethasone
**MM6**	Gain1q21,<br>del13q14	Kappa	6) carfilzomib+lenalidomide+dexamethasone+ASCT

Table S4. Patient characteristics related to Figure 4 and Supplementary Figure S5

Patient	Cytogenetics	M-protein	Stage at sampling	Lines of treatment
**MM7**	del13, gain11q	IgGK 4,2 g/L K: 762 mg/L	First line	-
**MM8**	normal	IgGL 39g/L	First line	-
**MM9**		IgGK 15g/L	Relapsed	1)Bortezomib+cyclophophamide+dexametasone-<br>2) bortezomib+thalidomide+dexamethasone +ASCT<br>3) Lenalidomide+bendamustine+ASCT<br>3) Lenalidomide<br>4)Daratumumab+cyclophsphamide+dexamethasone
**MM10**	normal	Lambda 706 mg/L	Relapsed	1)Bortezomib+ Lenalidomide+dexamethasone+ASCT<br>2) Daratumumab+Lenalidomide+dexamethasone
**MM11**		IgG 8,3 g/L K	Relapsed	1)Bortezomib+Lenalidomide+dexamethasone+ASCT+ Lenalidomide<br>2)Carfilzomib+dexamethasone + ASCT + carfilzomib
**MM12**		IgAL 8g/L mg/L	First line	-
**MM13**		IgG 14 g/L L 17mg/L	First line	-
**MM14**	de13q, gain 1q	IgGL 22g/L	Relapsed	1) Bortezomib+lenalidomide+dexamethasone+ASCT<br>2)Daratumumab+bortezomib+dexamethasone<br>3)Daratumumab+Lenalidomide+dexamethasone
**MM15**		IgGL	First line	-
**MM16**	normal	IgGK 10g/L K:14mg/L	Relapsed	1)Melphalane+prednisolone+thalidomide<br>2)Bortezomib+Lenalidomide+dexamethasone<br>3)Daratumumab+dexamethasone
**MM17**		IgGK 19g/L K:195mg/L	Relapsed	1) Melphalan +prednisolone
**MM18**	del13q	IgGK 36g/L	First line	-
**MM19**	gain 11q, del1p, gain 1q	IgGL 21g/L	First line	-
**MM20**	del13q, gain1q	IgGK 10g/L	Smouldering	-
**MM21**	normal	IgG 19g/L K: 184 mg/L	Smouldering	-
**MM22**		IgGK K:290 mg/L	Relapsed	1)Bortesomib+Lenalidomide+dexametahsone<br>2)Lenalidomide
**MM23**		IgGK	First line	
**MM24**		IgG 13.1 g/L	Smouldering	
**MM25**	gain11q13, gain 6p21	IgG 8g/L K: 188 mg/L	Relapsed	6 lines of treatment<br>line 6: Daratumumab + dexamethasone

ASCT: autologous stem cell transplant

References:

Alzamareh DF, Meednu N, Nandedkar-Kulkarni N, et al. Interferon activation in bone marrow long-lived plasma cells in systemic lupus erythematosus. Front Immunol 2024; 15: 1499551.
